# Cx43 hemichannels and panx1 channels contribute to ethanol-induced astrocyte dysfunction and damage

**DOI:** 10.1186/s40659-024-00493-2

**Published:** 2024-04-04

**Authors:** Gonzalo I. Gómez, Tanhia F. Alvear, Daniela A. Roa, Arantza Farias-Pasten, Sergio A. Vergara, Luis A. Mellado, Claudio J. Martinez-Araya, Juan Prieto-Villalobos, Claudia García-Rodríguez, Natalia Sánchez, Juan C. Sáez, Fernando C. Ortíz, Juan A. Orellana

**Affiliations:** 1 Institute of Biomedical Sciences, Faculty of Health Sciences, Universidad Autónoma de Chile, Santiago, Chile; 2 Departamento de Neurología, Escuela de Medicina and Centro Interdisciplinario de Neurociencias, Facultad de Medicina, Pontificia Universidad Católica de Chile, Marcoleta 391, Santiago, 8330024 Chile; 3 Instituto de Neurociencia, Centro Interdisciplinario de Neurociencia de Valparaíso, Facultad de Ciencias, Universidad de Valparaíso, Valparaíso, 2360102 Chile; 4 Department of Anatomy, Faculty of Medicine, Pontificia Universidad Católica de Chile, Santiago, Chile; 5 Departamento de Biología, Facultad de Química y Biología, Universidad de Santiago de Chile, Mechanisms of Myelin Formation and Repair Laboratory, Chacabuco 675, of. 212, Santiago, 8350347 Chile

**Keywords:** Hemichannels, Ethanol, Connexin-43, Pannexin-1, Astrocyte, Alcoholism and neuroinflammation

## Abstract

**Background:**

Alcohol, a widely abused drug, significantly diminishes life quality, causing chronic diseases and psychiatric issues, with severe health, societal, and economic repercussions. Previously, we demonstrated that non-voluntary alcohol consumption increases the opening of Cx43 hemichannels and Panx1 channels in astrocytes from adolescent rats. However, whether ethanol directly affects astroglial hemichannels and, if so, how this impacts the function and survival of astrocytes remains to be elucidated.

**Results:**

Clinically relevant concentrations of ethanol boost the opening of Cx43 hemichannels and Panx1 channels in mouse cortical astrocytes, resulting in the release of ATP and glutamate. The activation of these large-pore channels is dependent on Toll-like receptor 4, P2X7 receptors, IL-1β and TNF-α signaling, p38 mitogen-activated protein kinase, and inducible nitric oxide (NO) synthase. Notably, the ethanol-induced opening of Cx43 hemichannels and Panx1 channels leads to alterations in cytokine secretion, NO production, gliotransmitter release, and astrocyte reactivity, ultimately impacting survival.

**Conclusion:**

Our study reveals a new mechanism by which ethanol impairs astrocyte function, involving the sequential stimulation of inflammatory pathways that further increase the opening of Cx43 hemichannels and Panx1 channels. We hypothesize that targeting astroglial hemichannels could be a promising pharmacological approach to preserve astrocyte function and synaptic plasticity during the progression of various alcohol use disorders.

**Supplementary Information:**

The online version contains supplementary material available at 10.1186/s40659-024-00493-2.

## Background

Alcohol is the most commonly abused drug worldwide, and its consumption impairs life quality as it causes multiple chronic diseases and psychiatric states with serious negative health, societal, and economic consequences [1]. The high consumption of alcohol (6.4 L per capita per year) [2], its prevalence (43%), and the 3 million annual deaths resulting from its harmful use [2] highlight the need for a more profound knowledge of alcohol-related cellular targets. After crossing the blood-brain barrier, alcohol directly affects neurons by modulating NMDA receptors depending on the brain zone [3]. In addition, alcohol potentiates the function of GABA_A_ receptors [4] and glycine receptors [5]. However, the clinical trials testing the efficacy of most drugs against ethanol-related neuronal targets have been inadequate to draw definitive conclusions, and to date, they lack promising results [6].

An idea that gained ground in the last years argues that alcohol-induced brain alterations could depend partly on the miscommunication between neurons and one crucial glial population in the brain: the astrocyte [7, 8]. Astrocytes participate in the “tripartite synapse” -the cornerstone of the chemical synaptic transmission- in which they sense neural activity and respond to it by the intracellular free Ca^2+^ concentration ([Ca^2+^]_i_)-dependent release of bioactive molecules called “gliotransmitters” [9]. These messengers regulate the cerebral blood flow and exchange of energy-rich metabolites [10], contributing to the immune response and the homeostasis of the brain interstitial fluid [11]. During injury, astrocytes experience a long-lasting functional change referred to as “reactive astrogliosis”. The latter phenomenon includes cytoskeletal rearrangements, hypertrophy, metabolic alterations, and release of immunomodulatory mediators [12]. While this process limits injury and favors wound repair, it can be detrimental if it persists long enough to cause astroglial dysfunction and death, resulting in loss of the neuroprotective performance of astrocytes [13].

Ethanol, the main component of alcoholic beverages, elicits acute and long-lasting changes in reactive astrogliosis and different physiological aspects of astrocytes [14]. For example, mice exposed to ethanol over days or weeks show increased levels of GFAP [15, 16], and similar responses are observed in cultured astrocytes estimulated with ethanol over hours [15, 17]. In contrast, astrocyte cultures isolated from prenatally ethanol exposed mice exhibit a decrease in GFAP levels [18, 19]. On the other hand, acute (minutes) and long-term (hours-days) incubation with ethanol increases the expression of iNOS and COX-2 and the production of pro-inflammatory cytokines and chemokines in cultured astrocytes [20–22]. These responses take place concomitantly with the activation of the nuclear factor NF-κB and subsequent redox imbalance, mitochondrial dysfunction and cell death [22–24]. Additionally, ethanol acutely induces [Ca^2+^]_i_ transients [25, 26] and disturbs astrocyte-astrocyte communication along with astroglial gliotransmission in cell cultures [27, 28]. The mechanisms driving these changes and the significant repercussions of astroglial dysfunction on ethanol-induced brain abnormalities remain unclear.

Multiple studies have described that large-pore channels formed by connexins and pannexins [29] perturb astrocytic function in various brain diseases and pathological conditions [30–39]. Connexins encompass a family of membrane proteins that oligomerize around a central pore to form hexameric conduits called connexons [40]. The docking of two connexons, also known as hemichannels, from adjacent cells bridges the extracellular space, creating a gap junction channel (GJC). The arrangement of a variable number of these channels at specialized membrane areas, called gap junctions, allows direct communication between the cytosolic compartments of contacting cells [40]. Hemichannels at non-junctional membranes act as large pore channels selective for ions and molecules, facilitating the exchange between the cytosol and the extracellular space of substances below 1.5 kDa [41].

Over two decades ago, Panchin and colleagues identified the mammalian counterparts of innexins, the gap junction proteins found in invertebrates [42]. They named this family “pannexins,” suggesting the existence of a distinct type of GJC in vertebrate cells. Pannexins embrace a three-member family of membrane proteins (Panx1-3) that form pannexons, that ‒like undocked connexons‒ communicate the cytoplasm with the interstitium [43]. However, the absence of consistent data on exogenous pannexin cell-cell channels [44–48] and the occurrence of negative results in cells endogenously expressing pannexins led multiple research groups to dismiss the notion that pannexins constitute GJCs under normal physiological conditions [49, 50]. Recently, the systematic study of macroscopic and single-channel transjunctional currents revealed that endogenously expressed human Panx1 forms cell–cell channels with unique properties resembling those described in innexin-based GJCs [51].

Astrocytes exhibit functional hemichannels and pannexons formed mainly by connexin 43 (Cx43) and Panx1, respectively, as has been demonstrated in in vitro and ex vivo preparations [32, 52–54]. Cellular signaling via the opening of these astrocytic large-pore channels underpins synaptic transmission and plasticity [55–59], as well as memory consolidation and behavior [60–63]. Despite the above, mounting evidence argues that persistent and increased opening of Cx43 hemichannels and Panx1 channels could affect the interactive partnership between astrocytes and neurons, with potentially harmful consequences for adequate brain function [64–67]. Recently, our laboratory described that intermittent intraperitoneal injections with ethanol increase the opening of Cx43 hemichannels and Panx1 channels in astrocytes [16]. However, whether ethanol directly affects astroglial hemichannels/pannexons or modulates them indirectly through immunomodulatory factors from microglia remains a critical question awaiting resolution.

Here, we show that ethanol augments the activity of Cx43 hemichannels and Panx1 channels in mouse cortical astrocytes, leading to the release of ATP and glutamate. The effect on these large-pore channels depended on the activation of Toll-like receptor 4 (TLR4), P2X7 receptors, IL-1β and TNF-α signaling, the p38 mitogen-activated protein kinase (p38 MAPK) and the inducible nitric oxide (NO) synthase (iNOS). Notably, the ethanol-induced activity of hemichannels and pannexons resulted in alterations in cytokine secretion, NO production, gliotransmitter release, astrocyte reactivity and survival. As a result, these changes induced by ethanol significantly impact astrocyte function, reactivity, and viability, emphasizing the complex interplay between ethanol exposure and astroglial dynamics in the brain.

## Results

### Ethanol elevates the activity of Cx43 hemichannels and Panx1 channels in cultured astrocytes

Ethanol increases the inflammatory profile of astrocytes [20, 21] and impairs their physiology, including [Ca^2+^]_i_ homeostasis, intercellular communication and gliotransmission [25–28]. On the other hand, mounting evidence has documented that Cx43 hemichannels and Panx1 channels disturb astrocytic function in multiple brain diseases and pathological conditions [30–39]. However, whether ethanol could modulate the activity of these channels in primary cortical astrocytes remains unknown. We assessed the functional state of hemichannels and pannexons in astrocytes by measuring the rate of ethidium (Etd, charge^1+^) uptake, a dye entering healthy cell cytoplasm through plasma membrane channels with large pores [68]. Etd becomes fluorescent upon intercalation with base pairs of DNA and RNA, reflecting channel activity.

**Fig. 1:**
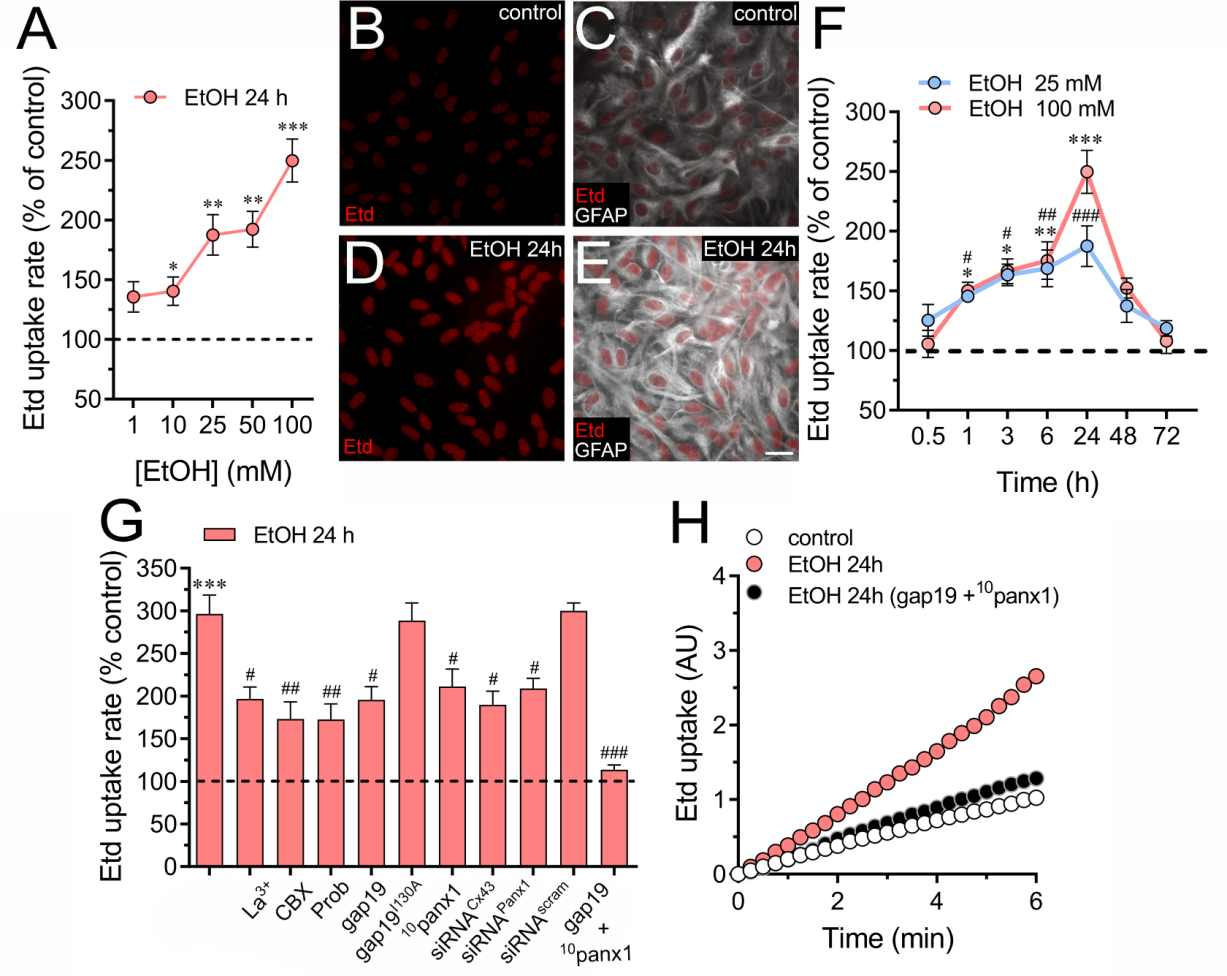
Ethanol increases the activity of Cx43 hemichannels and Panx1 channels in cultured astrocytes. (**A**) Averaged Etd uptake rate (AU/min) normalized with the control condition (dashed line) by astrocytes treated for 24 h with different concentrations of ethanol (red circles). *p < 0.05, **p < 0.005, ***p < 0.0001, ethanol treatment compared to control conditions (one-way ANOVA followed by Tukey’s post-hoc test). (**B**-**E**) Representative immunofluorescence images depicting Etd and GFAP staining from dye uptake measurements (10 min exposure to Etd) in astrocytes under control conditions (**B-C**) or treated for 24 h with 100 mM ethanol (**D-E**). (**F**) Averaged Etd uptake rate (AU/min) normalized with the control condition (dashed line) by astrocytes treated for several time periods with ethanol at two concentrations: 25 mM (blue circles) or 100 mM (red circles). *p < 0.05, **p < 0.005, ***p < 0.0001, 100 mM ethanol treatment compared to control conditions; ^#^*p* < 0.05, ^##^*p* < 0.005, ^###^*p* < 0.0001, 25 mM ethanol treatment compared to control conditions (one-way ANOVA followed by Tukey’s post-hoc test). (**G**) Averaged Etd uptake rate (AU/min) normalized with control condition (dashed line) by astrocytes treated for 24 h with 100 mM ethanol alone or in combination with the following blockers: 200 µM La^3+^, 5 µM carbenoxolone (CBX), 500 µM Probenecid (Prob), 50 µM gap19, 50 µM gap19^I130A^, 50 µM ^10^panx1, siRNA^Cx43^, siRNA^Panx1^; siRNA^scrb^ and 50 µM gap19 + 50 µM ^10^panx1. ***p < 0.0001, ethanol compared to control; ^#^*p* < 0.05, ^##^*p* < 0.005; ^###^*p* < 0.001; effect of pharmacological agents compared to ethanol treatment (one-way ANOVA followed by Tukey’s post-hoc test). (**H**) Time-lapse measurements of Etd uptake by astrocytes under control conditions (white circles) or treated for 24 h with 100 mM ethanol alone (red circles) or in combination with 50 µM gap19 + 50 µM ^10^panx1 (black circles). Data were obtained from at least three independent experiments with three or more repeats each one (≥ 30 cells analyzed for each repeat). Calibration bar = 30 μm

Ethanol treatment led to a concentration-dependent augment in astrocytic Etd uptake, this response being significant at 10 mM, with 100 mM representing the concentration that elicited the maximum effect (Fig. 1A). Temporal analysis of this response revealed a sustained increase in Etd uptake compared to control conditions, reaching its peak after 24 h, followed by a gradual decline (Fig. 1B-F). With this in mind, we opted for the treatment of 100 mM ethanol for 24 h in further experiments. As Cx43 hemichannels and Panx1 channels significantly contribute to dye influx in astrocytes [52, 69], we delved into their potential involvement in ethanol-induced Etd uptake. The involvement of Cx43 hemichannels in ethanol-dependent Etd uptake was investigated using a nonselective inhibitor of connexin hemichannels, La^3+^ [70], and the specific mimetic peptide known to block Cx43 hemichannels, gap19 [71, 72]. Both La^3+^ (200 µM) and gap19 (50 µM) partially reduced ethanol-induced Etd uptake in astrocytes (Fig. 1G). Interestingly, the inactive form of gap19 (gap19^I130A^) showed no inhibitory effect (Fig. 1G). In contrast, Cx43 knockdown with siRNA prevented ethanol-induced Etd uptake, mirroring the effects observed with La^3+^ and gap19 (Fig. 1G). To scrutinize the role of Panx1 channels, we pharmacologically inhibited them with 5 µM carbenoxolone (CBX), probenecid (500 µM) and the mimetic peptide ^10^panx1 (50 µM) [73]. All of these interventions attenuated the Etd uptake observed in ethanol-treated astrocytes (Fig. 1G). Similar results were observed upon downregulation of Panx1 with siRNA^Panx1^ (Fig. 1G). Importantly, the concurrent use of gap19 and ^10^panx1 completely abolished ethanol-induced Etd uptake (Fig. 1H), strongly indicating that ethanol enhances the opening of Cx43 hemichannels and Panx1 channels in astrocytes. We also noted that astrocytes treated with ethanol exhibited increased levels of GFAP (Fig. 1E).

**Fig. 2:**
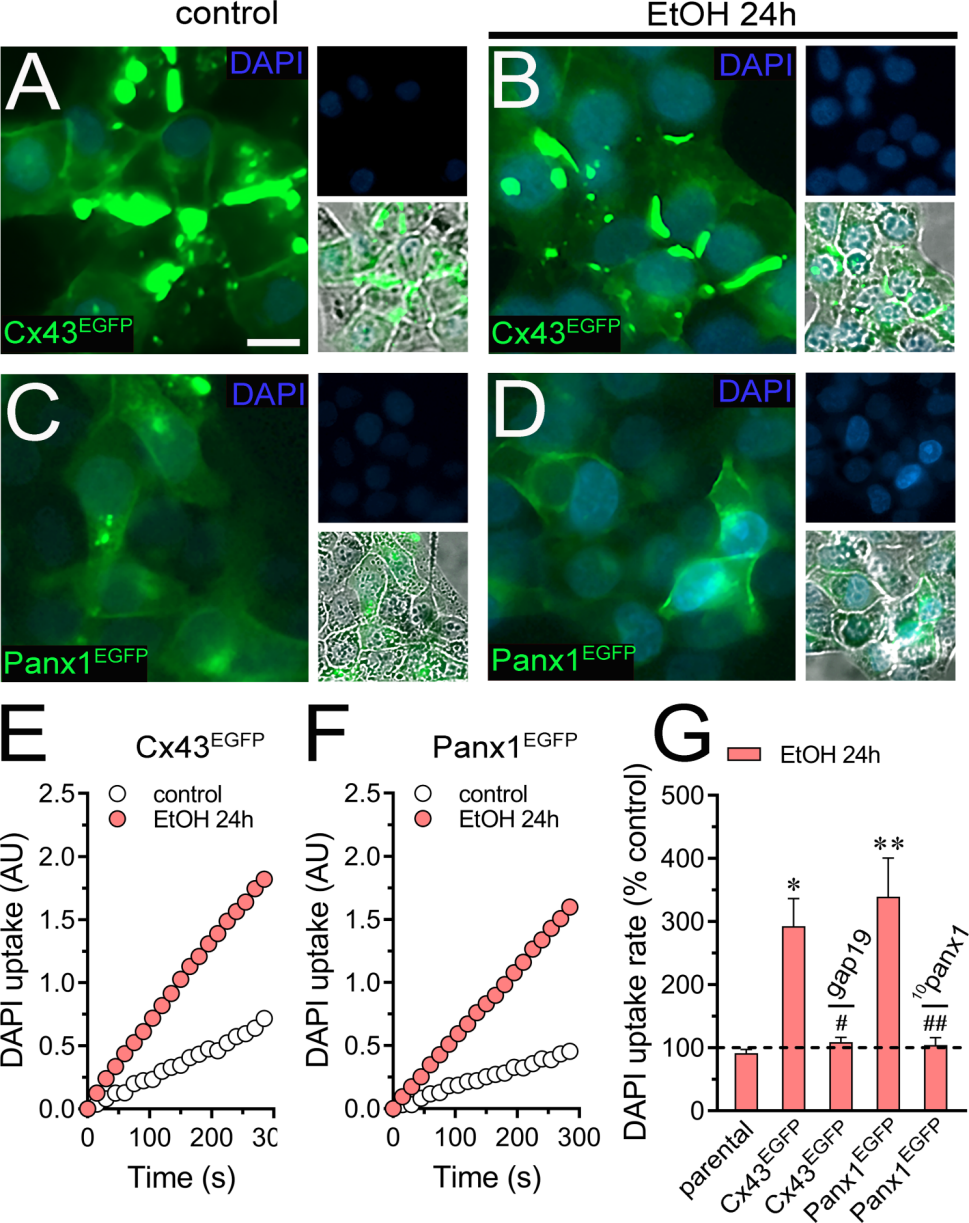
Ethanol increases the activity of large-pore channels in HeLa cells transfected with Cx43 or Panx1. (**A**-**D**) Representative images depicting Cx43^EGFP^ (green), Panx1^EGFP^ (green) and DAPI (blue, 5 µM and 10 min of exposure) labeling by HeLa-Cx43^EGFP^ (**A-B**) and HeLa-Panx1 ^EGFP^ (**C-D**) cells under control conditions or treated for 24 h with 100 mM ethanol. Insets at the right of each panel depict the respective DAPI labeling alone (top) or plus the phase view merged with EGFP (bottom). (**E-F**) Time-lapse measurements of DAPI uptake by HeLa-Cx43^EGFP^ (**E**) and HeLa-Panx1 ^EGFP^ (**F**) cells under control conditions (white circles) or treated for 24 h with 100 mM ethanol (red circles). (**G**) Averaged DAPI uptake rate (AU/min) normalized with the control condition (dashed line) by parental HeLa, HeLa-Cx43^EGFP^ and HeLa-Panx1 ^EGFP^ cells treated for 24 h with 100 mM ethanol. In some experiments, HeLa-Cx43^EGFP^ or HeLa-Panx1 ^EGFP^ cells were treated for 24 h with 100 mM ethanol plus 50 µM gap19 or 50 µM ^10^panx1, respectively. *p < 0.05, **p < 0.01, ethanol treatment compared to control conditions (two-way ANOVA followed by Tukey’s post-hoc test). Data were obtained from at least three independent experiments with three or more repeats each one (≥ 20 cells analyzed for each repeat). Calibration bar = 15 μm

To investigate whether ethanol-induced channel activity extends to a different cell type and if this effect can be replicated in an exogenous expression system, we transfected HeLa cells with Cx43 or Panx1 tagged with EGFP. These transfected cells express hemichannels and pannexons at the cell surface, allowing them to uptake and release small molecules, including dyes commonly used to assay channel activity [74–76]. Following treatment with 100 mM ethanol for 24 h, both HeLa-Cx43^EGFP^ and HeLa-Panx1^EGFP^ cells exhibited a substantial ∽ 3 and 3.5-fold increase in the uptake of DAPI (charge^2+^) (Fig. 2A-G), another well-known dye that permeates large-pore channels [74, 76]. Relevantly, the same abovementioned stimulus did not alter the DAPI uptake in HeLa-parental cells (non-transfected), revealing that ethanol increases specifically the activity of Cx43 hemichannels and Panx1 channels in HeLa cells (Fig. 2G). Confirming these data, 50 µM gap19 or 50 µM ^10^panx1 completely blunted the ethanol-induced DAPI uptake in HeLa-Cx43^EGFP^ and HeLa-Panx1^EGFP^ cells, respectively (Fig. 2G).

### Ethanol does not affect total and plasma membrane levels of Cx43 and Panx1

**Fig. 3:**
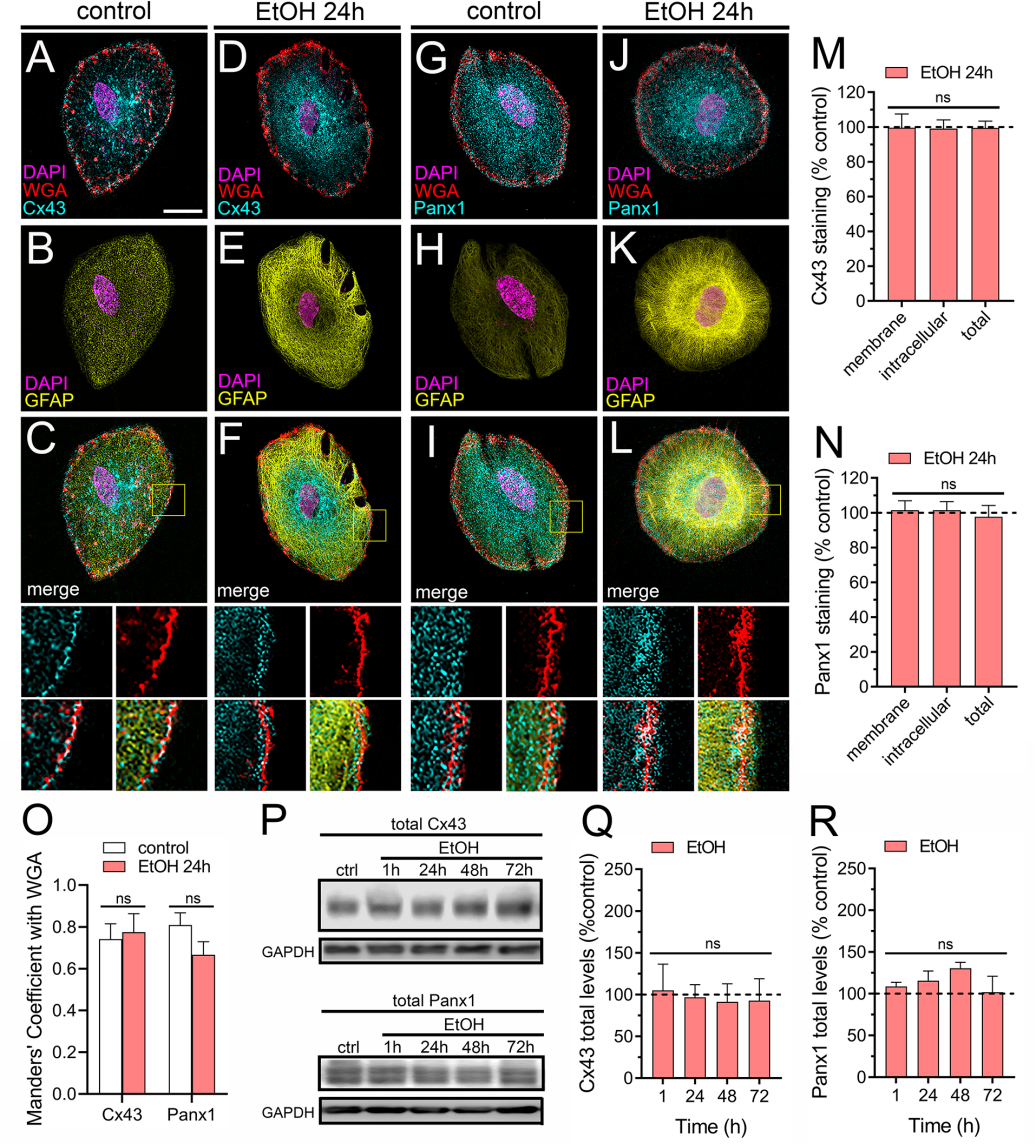
Ethanol does not modulate levels and plasma membrane distribution of Cx43 and Panx1 in cultured astrocytes. (**A-L**) Representative confocal images depicting Cx43 (cyan, left panel) or Panx1 (cyan, right panel) immunostaining in combination with DAPI (magenta) and WGA (red) labeling by astrocytes under control conditions (**A-C** and **G-I**) or treated for 24 h with 100 mM ethanol (**D-F** and **J-L**). The size of the z-step acquisition for all images was 0.3 μm. Calibration Bar: 10 μm. Insets: 2X magnification of the indicated area of panels C, F, I and L. (**M**-**N**) Quantification of membrane, intracellular and total staining of Cx43 (M) and Panx1 (N) normalized to control conditions (dashed line) by astrocytes treated for 24 h with 100 mM ethanol. (**O**) Quantification of Manders’ overlap coefficient for Cx43 or Panx1 with WGA by astrocytes under control conditions (white bars) or treated for 24 h with 100 mM ethanol (red bars). (**P**) Total Cx43 (upper panel) and Panx1 (bottom panel) levels by astrocytes under control conditions or treated for 1, 24, 48–72 h with 100 mM ethanol. Total levels of each analyzed band were normalized according to the levels of GADPH detected in each lane. (**Q-R**) Quantification of total levels of Cx43 (**Q**) and Panx1 (**R**) normalized to control (dashed line) in astrocytes treated for 1, 24, 48–72 h with 100 mM ethanol. Averaged data were obtained from three independent experiments

The activity of hemichannels and pannexons can be profoundly shaped by fluctuations in their individual open probability, conductance characteristics, selectivity, and the number of these channels embedded within the cell membrane. Channel-dependent dye uptake has been associated with heightened surface levels of Cx43 and Panx1 across various cell types [77–79], or an elevation in open probability without concurrent alterations in the overall protein content on the cell surface [80]. Thus, we used confocal microscopy to scrutinize whether ethanol-induced Etd uptake correlates with modifications in the overall and/or surface concentrations of Cx43 and Panx1 in subconfluent astrocytes. Colocalization analysis using the membrane marker wheat germ agglutinin (WGA) demonstrated that surface levels of Cx43 and Panx1 remained unaltered after stimulating astrocytes with ethanol for 24 h (Fig. 3A-N). Consistent with these findings, we did not observe any changes in Manders’ coefficients for the colocalization of Cx43 or Panx1 with WGA. (Fig. 3O). Moreover, western blot experiments showed that a 24-hour ethanol treatment had no impact on the total levels of both Cx43 and Panx1 in astrocytes. (Fig. 3P-R). These insights indicate that ethanol-induced activity of Cx43 hemichannels and Panx1 channels is likely not driven by changes in channel abundance on the cell surface.

### Ethanol-induced Cx43 hemichannel activity depends on TLR4 and IL-1β/TNF-α/p38 MAPK/iNOS-dependent signaling

**Fig. 4:**
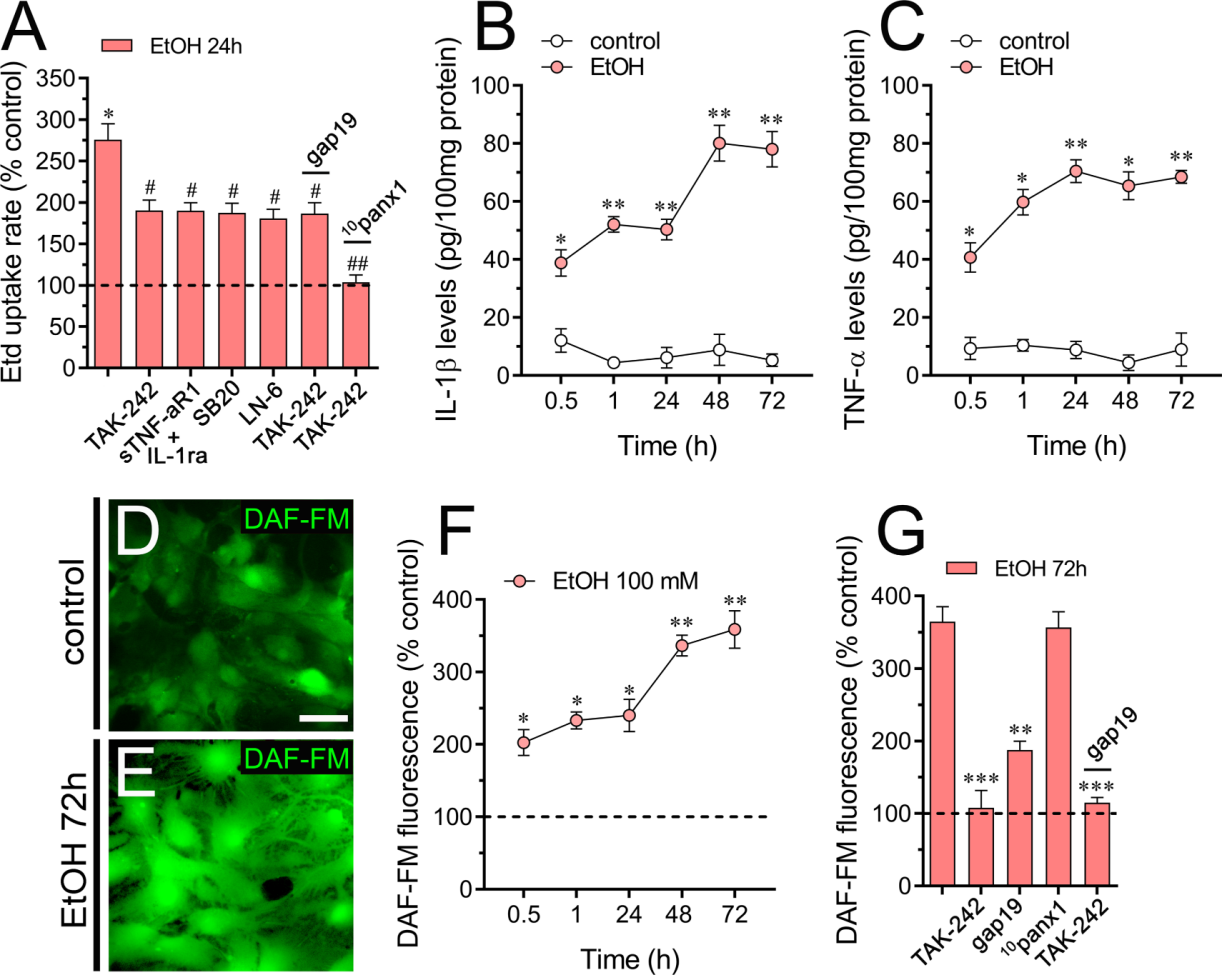
Ethanol-induced Cx43 hemichannel activity depends on TLR4 and IL-1β/TNF-α/p38 MAPK/iNOS-dependent signaling. (**A**) Averaged Etd uptake rate (AU/min) normalized with control condition (dashed line) by astrocytes treated for 24 h with 100 mM ethanol alone or in combination with the following agents: 0.5 µM TAK-242, 100 ng/mL of IL-1ra + 100 ng/mL of sTNF-αR1, 1 µM SB203580, 1 µM L-N6, 20 nM 0.5 µM TAK-242 + 50 µM gap19 or 0.5 µM TAK-242 + 50 µM ^10^panx1. *p < 0.005, ethanol treatment compared to control conditions; ^#^*p* < 0.01; ^##^*p* < 0.005; effect of pharmacological agents compared to ethanol treatment (one-way ANOVA followed by Tukey’s post-hoc test). Data were obtained from at least three independent experiments with three or more repeats each one (≥ 30 cells analyzed for each repeat). (**B-C**) Averaged data of IL-1β (**B**) and TNF-α (**C**) released by astrocytes under control conditions (white circles) or treated for several time periods with 100 mM ethanol (red circles). *p < 0.05, **p < 0.01, ethanol treatment compared to control conditions (one-way ANOVA followed by Tukey’s post-hoc test). (**D-E**) Representative fluorescence micrographs of basal NO production (DAF-FM, green) by astrocytes under control conditions (**D**) or treated for 24 h with 100 mM ethanol (**E**). Calibration Bar: 20 μm. (**F**) Average DAF-FM fluorescence normalized to control conditions (dashed line) by astrocytes treated with 100 mM ethanol for several time periods. *p < 0.05, **p < 0.005, ethanol treatment compared to control conditions (one-way ANOVA followed by Tukey’s post-hoc test). (**G**) Average DAF-FM fluorescence normalized to control conditions (dashed line) by astrocytes treated for 72 h with 100 mM ethanol alone or in combination with the following agents: 0.5 µM TAK-242, 50 µM gap19, 50 µM ^10^panx1 or 0.5 µM TAK-242 + 50 µM gap19. ***p < 0.005, **p < 0.001, effect of pharmacological agents compared to ethanol treatment (one-way ANOVA followed by Tukey’s post-hoc test)

Earlier studies have established that ethanol amplifies the inflammatory characteristics of astrocytes by engaging the TLR4 and/or IL-1RI signaling [21]. Moreover, a growing body of evidence suggests that the activation of astrocytic Cx43 hemichannels during pro-inflammatory conditions relies on the generation of TNF-α and IL-1β, along with the activation of both p38 MAPK and iNOS, leading to the subsequent production of NO [34, 35, 81]. In this scenario, we investigated their potential role in ethanol-induced Etd uptake in astrocytes. Pre-treatment with TAK-242 (300 nM), a TLR4 inhibitor, substantially but not totally reduced ethanol-induced Etd uptake (Fig. 4A). Likewise, a soluble TNF-α receptor that binds TNF-α (sTNF-aR1) and an IL-1β receptor antagonist (IL-1ra) significantly suppressed the Etd uptake triggered by ethanol (Fig. 4A). Applying the p38 MAPK inhibitor SB202190 (1 µM) or blocking iNOS with 5 µM L-N6 evoked similar counteracting responses on ethanol-induced Etd uptake (Fig. 4A).

Interestingly, the Cx43 hemichannel blocker, gap19, showed no additional inhibitory effect on ethanol-induced Etd uptake when astrocytes were treated with TAK-242 (Fig. 4A). The latter suggests that Cx43 hemichannel-mediated response to EtOH depends exclusively on TLR4 activation. In contrast, the Panx1 channel blocker, ^10^panx1, demonstrated an additive inhibitory effect when astrocytes were exposed to TAK-242 (Fig. 4A). In instances of disease, astrocytes may release substantial amounts of pro-inflammatory cytokines, impacting astrocyte homeostasis at multiple levels: molecular, morphological, and functional [82]. Notably, the exposure of astrocytes to IL-1β and TNF-α has been observed to induce the opening of Cx43 hemichannels [30, 81]. Considering that sTNF-aR1 and IL-1ra significantly reduced the ethanol-induced Etd uptake by astrocytes (Fig. 4A), we explored whether ethanol could affect the release of IL-1β and TNF-α in our system. Exposure to ethanol for 30 min triggered a strong surge in the release of IL-1β and TNF-α in astrocytes (Fig. 4B and C). Furthermore, ethanol treatment led to a sustained elevation in the release of both cytokines over time, reaching a plateau after 24 and 48 h for IL-1β and TNF-α, respectively (Fig. 4B and C).

Astrocytes are known to undergo p38 MAPK activation through IL-1β/TNF-α signaling, culminating in the expression of iNOS and subsequent NO production [83, 84]. The latter and the robust counteraction of ethanol-mediated Etd uptake in astrocytes by inhibiting iNOS with LN-6, prompted us to examine the possible impact of ethanol on NO production in these cells. DAF-FM fluorescence imaging unveiled that ethanol caused a time-dependent rise in NO levels, stabilizing after 48–72 h of treatment (Fig. 4D-F). Crucially, TAK-242 completely suppressed the ethanol-induced production of NO following 72 h of treatment (Fig. 4G). Of note, gap19 strongly (though not completely) reduced the ethanol-induced production of NO, while blocking Panx1 channels with ^10^panx1 failed to elicit equivalent inhibitory effects (Fig. 4G). Significantly, TAK-242 exhibited an additional inhibitory effect on the reduction of ethanol-induced NO production caused by gap19. (Fig. 4G). Collectively, these findings emphasize that within the TLR4-mediated cascade leading to enhanced NO production by ethanol, the signaling predominantly hinges on Cx43 hemichannels rather than Panx1.

### Ethanol induces the release of ATP and glutamate through different pathways

**Fig. 5:**
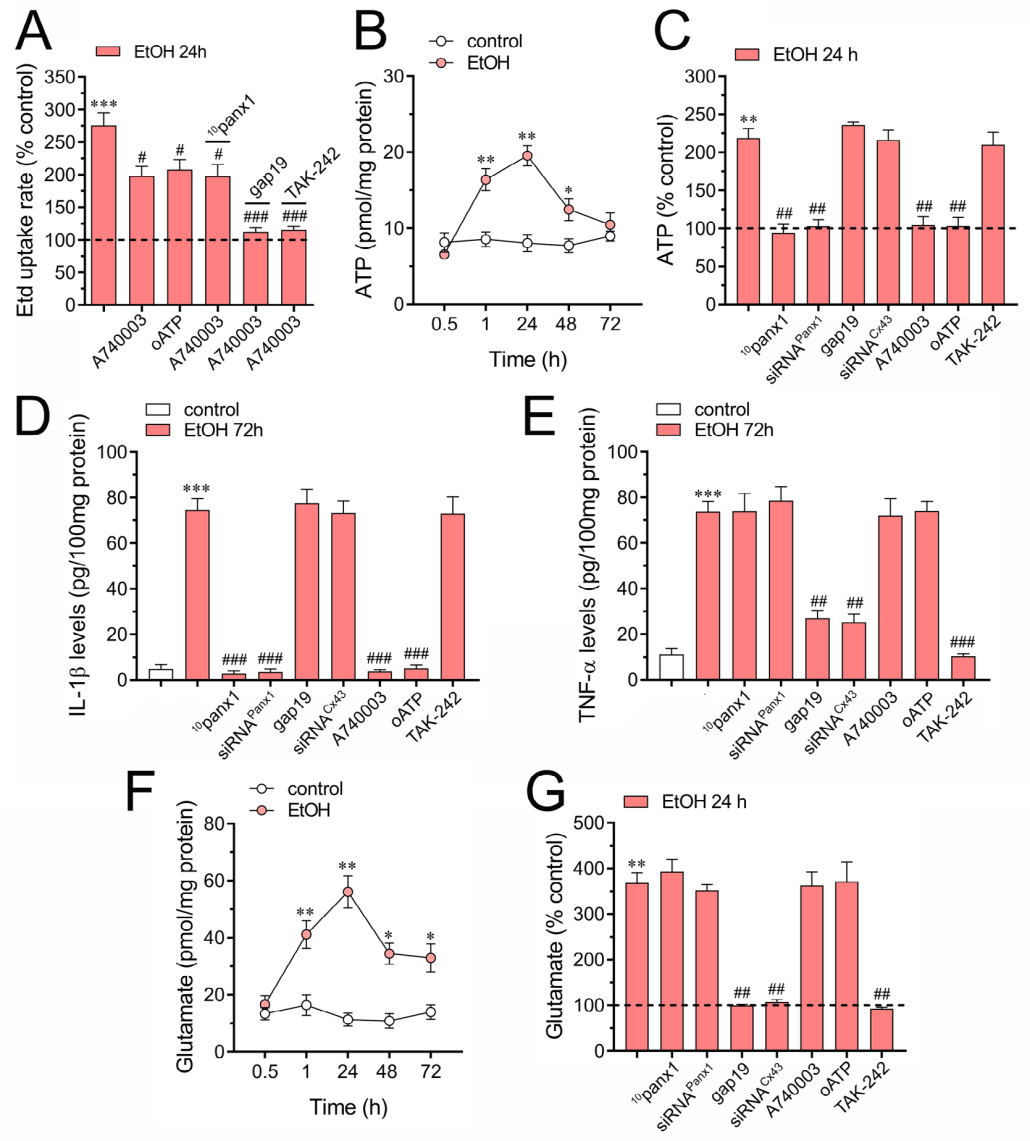
The ethanol-induced activation of hemichannels and pannexons triggers the release of ATP and glutamate by different pathways in cultured astrocytes. (**A**) Averaged Etd uptake rate (AU/min) normalized with control condition (dashed line) by astrocytes treated for 24 h with 100 mM ethanol alone or in combination with the following agents: 200 nM A740003, 200 µM oATP, 200 nM A740003 + 50 µM ^10^panx1, 200 nM A740003 + 50 µM gap19 or 200 nM A740003 + 0.5 µM TAK-242. ***p < 0.001, ethanol treatment compared to control conditions; ^#^*p* < 0.05; ^###^*p* < 0.005; effect of pharmacological agents compared to ethanol treatment (one-way ANOVA followed by Tukey’s post-hoc test). (**B**) Averaged data of ATP release by astrocytes under control conditions (white circles) or treated with 100 mM ethanol for different time periods (red circles). *p < 0.05, **p < 0.01, ethanol treatment compared to control conditions (one-way ANOVA followed by Tukey’s post-hoc test). (**C**) Averaged data of ATP release by astrocytes under control conditions (dashed line) or treated for 24 h with 100 mM ethanol alone or in combination with the following agents: 50 µM ^10^panx1, siRNA^Panx1^, 50 µM gap19, siRNA^Cx43^, 200 nM A740003, 200 µM oATP or 0.5 µM TAK-242. **p < 0.01, ethanol treatment compared to control conditions; ^##^*p* < 0.01; effect of pharmacological agents compared to ethanol treatment (one-way ANOVA followed by Tukey’s post-hoc test). (**D-E**) Averaged data of IL-1β (**D**) and TNF-α (**E**) release by astrocytes under control conditions (white bar) or treated for 72 h with 100 mM ethanol (red bars) or in combination with the following agents: 50 µM ^10^panx1, siRNA^Panx1^, 50 µM gap19, siRNA^Cx43^, 200 nM A740003, 200 µM oATP or 0.5 µM TAK-242. ***p < 0.005, ethanol treatment compared to control conditions; ^###^*p* < 0.005; effect of pharmacological agents compared to ethanol treatment (one-way ANOVA followed by Tukey’s post-hoc test). (**F**) Averaged data of glutamate release by astrocytes under control conditions (white circles) or treated with 100 mM ethanol for different time periods (red circles). *p < 0.05, **p < 0.01, ethanol treatment compared to control conditions (one-way ANOVA followed by Tukey’s post-hoc test). (**G**) Averaged data of glutamate release by astrocytes under control conditions (dashed line) or treated for 24 h with 100 mM ethanol alone or in combination with the following agents: 50 µM ^10^panx1, siRNA^Panx1^, 50 µM gap19, siRNA^Cx43^, 200 nM A740003, 200 µM oATP or 0.5 µM TAK-242. **p < 0.01, ethanol treatment compared to control conditions; ^##^*p* < 0.01; effect of pharmacological agents compared to ethanol treatment (one-way ANOVA followed by Tukey’s post-hoc test). Data were obtained from at least three independent experiments with three or more repeats each one (≥ 30 cells analyzed for each repeat)

In neuropathological scenarios, the release of ATP often accompanies the situation, acting as a versatile danger signal. This signal triggers reactive astrogliosis and recruits microglia and other peripheral immune cells, thereby increasing the susceptibility of neurons to damage [85]. Since Cx43 hemichannels and Panx1 channels facilitate the release of ATP in astrocytes [52, 53], and considering that ATP activates these channels by increasing the influx of Ca^2+^ via P2X7 receptors [86, 87], we evaluated whether these receptors play a role in ethanol-induced channel activity. Blocking P2X7 receptors with 200 nM A740003 or 200 µM oxidized ATP (oATP) partially decreased ethanol-induced Etd uptake (Fig. 5A). Of note, the Panx1 channel blocker, ^10^panx1, exhibited no additional counteracting effect on ethanol-induced Etd uptake when astrocytes were treated with A740003 (Fig. 5A). In contrast, suppressing TLR4 or Cx43 hemichannel activity with TAK-242 or gap19, respectively, showed an additive blocking effect when astrocytes were exposed to A740003 (Fig. 5A).

Luciferin/luciferase assays indicated that ethanol triggered a bell-shaped increase in ATP release over time, reaching its peak around 24 h of treatment (Fig. 5B). Importantly, ^10^panx1 or downregulation of Panx1 with siRNA^Panx1^ totally abrogated ethanol-induced release of ATP (Fig. 5C). In contrast, pharmacological inhibition of Cx43 hemichannels (using gap19) or downregulation of Cx43 (with siRNA^Cx43^) failed in cause a similar counteracting response (Fig. 5C). Notably, the inhibition of P2X7 receptors (using A740003 or oATP) but not TLR4 (using TAK-242) fully reduced ethanol-induced release of ATP (Fig. 5C). Prior research has established that hemichannels and pannexons contribute to the inflammasome-mediated production of IL-1β in diverse cell types [88, 89], including astrocytes [90]. In our system, the maximal response to the ethanol-induced release of IL-1β after 72 h of treatment was suppressed entirely by pharmacological blockade of Panx1 channels or P2X7 receptors and downregulation of Panx1 (Fig. 5D). On the contrary, gap19 or TAK-242 did not prevent the ethanol-induced release of IL-1β (Fig. 5D). Relevantly, the ethanol-induced release of TNF-α after 72 h strongly reduce upon downregulation of blockade of Cx43 hemichannels or TLR4 (Fig. 5E). In contrast, A740003, oATP, ^10^panx1 or siRNA^Panx1^ failed in induce a similar response (Fig. 5E). Altogether, these findings suggest that P2X7 receptors mediate the ethanol-induced activity of Panx1 channels and subsequent release of ATP and IL-1β. On the other hand, it seems that TLR4 and further activation of Cx43 hemichannels participate in the long-lasting release of TNF-α from astrocytes.

Reactive astrogliosis could potentially result in the excitotoxic release of glutamate [91], with one of the implicated mechanisms being the activity of hemichannels and pannexons [64]. Under this premise, we investigated whether ethanol influences the release of glutamate from astrocytes. Similar to our findings with ATP, ethanol triggered a bell-shaped increase in glutamate release over time, with the peak occurring around 24 h of treatment (Fig. 5F). Notably, neither ^10^panx1 nor the downregulation of Panx1 with siRNA^Panx1^ proved effective in preventing ethanol-induced release of glutamate (Fig. 5G). In contrast, both pharmacological inhibition of Cx43 hemichannels (with gap19) and downregulation of Cx43 (with siRNA^Cx43^) significantly reduced the glutamate release induced by ethanol (Fig. 5G). Importantly, the complete abolishment of ethanol-induced glutamate release was observed with the inhibition of TLR4 (using TAK-242), but not with P2X7 receptors (using A740003 or oATP) (Fig. 5G). In summary, these observations suggest that ethanol induces the release of ATP and glutamate in astrocytes through distinct and separate mechanisms, specifically, Panx1 channels and Cx43 hemichannels, respectively.

### Inhibition of hemichannels and pannexons reduces the ethanol-induced astroglial reactivity and cell death

**Fig. 6:**
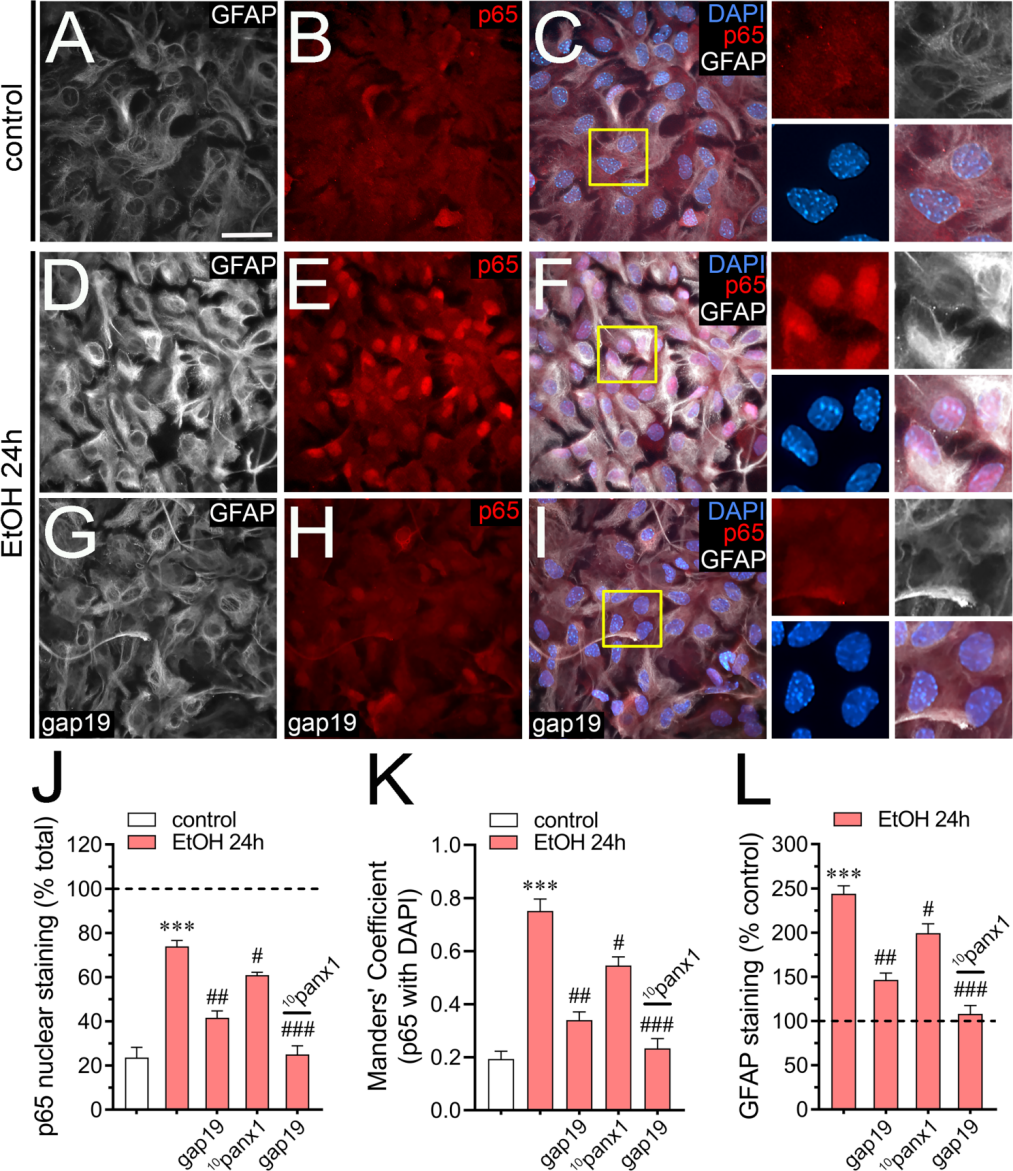
Ethanol boosts the expression of GFAP and NF-κB p65 by a mechanism involving the activation of hemichannels and pannexons in cultured astrocytes. (**A-I**) Representative fluorescence micrographs of GFAP (white), NF-κB p65 (red) and DAPI (blue) labeling by astrocytes under control conditions (**A-C**) or treated for 24 h with 100 mM ethanol alone (**D-F**) or in combination with 50 µM gap19 (**G-I**). Insets: 2X magnification of the indicated area of panels C, F and I. (**J**) Quantitation of NF-κB p65 nuclear staining by astrocytes under control conditions (white bars) or treated for 24 h with 100 mM ethanol alone (red bars) or in combination with 50 µM gap19, 50 µM ^10^panx1 or 50 µM gap19 plus 50 µM ^10^panx1. (**K**) Quantification of Manders’ overlap coefficient for NF-κB p65 with DAPI by astrocytes under control conditions (white bars) or treated for 24 h with 100 mM ethanol alone (red bars) or in combination with 50 µM gap19, 50 µM ^10^panx1 or 50 µM gap19 plus 50 µM ^10^panx1. (**L**) Quantitation of GFAP staining normalized to control conditions (dashed line) by astrocytes treated for 24 h with 100 mM ethanol alone (red bars) or in combination with 50 µM gap19, 50 µM ^10^panx1 or 50 µM gap19 plus 50 µM ^10^panx1. ***p < 0.0005, ethanol treatment compared to control conditions, ^#^*p* < 0.05, ^##^*p* < 0.001, effect of pharmacological agents compared to ethanol treatment (one-way ANOVA followed by Tukey’s post-hoc test). Data were obtained from at least three independent experiments with three or more repeats each one (≥ 20 cells analyzed for each repeat). Calibration bar = 80 μm

We recently showed that in vivo ethanol administration induces neuroinflammation and increases astrocyte reactivity [16]. Similar results have been observed in cultured astrocytes treated with ethanol [20, 21]. Notably, the uncontrolled opening of hemichannels and pannexons appears crucial for causing cell damage in astrocytes [35, 92]. In light of this, we delved into whether ethanol impacts the levels of NF-κB p65 and GFAP, along with astrocyte survival. After 24 h of ethanol exposure, there was a notable increase in NF-κB p65 staining within the nucleus, alongside heightened GFAP labeling in astrocytes (Fig. 6A-L, see also Fig. 1B-C). Western blot analysis showed that the ethanol-induced rise in GFAP levels occurred after 24 h of treatment, persisting at elevated levels until 72 h of treatment (Supplementary Fig. 1). The inhibition of Cx43 hemichannels with gap19 robustly prevented the ethanol-induced increase in nuclear NF-κB p65 and GFAP levels while blocking Panx1 channels (using ^10^panx1) was also effective but to a lesser extent (Fig. 6J-L). Relevantly, when both channels were blocked, the treatment with ethanol did not change nuclear NF-κB p65 and GFAP levels (Fig. 6J-L).

**Fig. 7:**
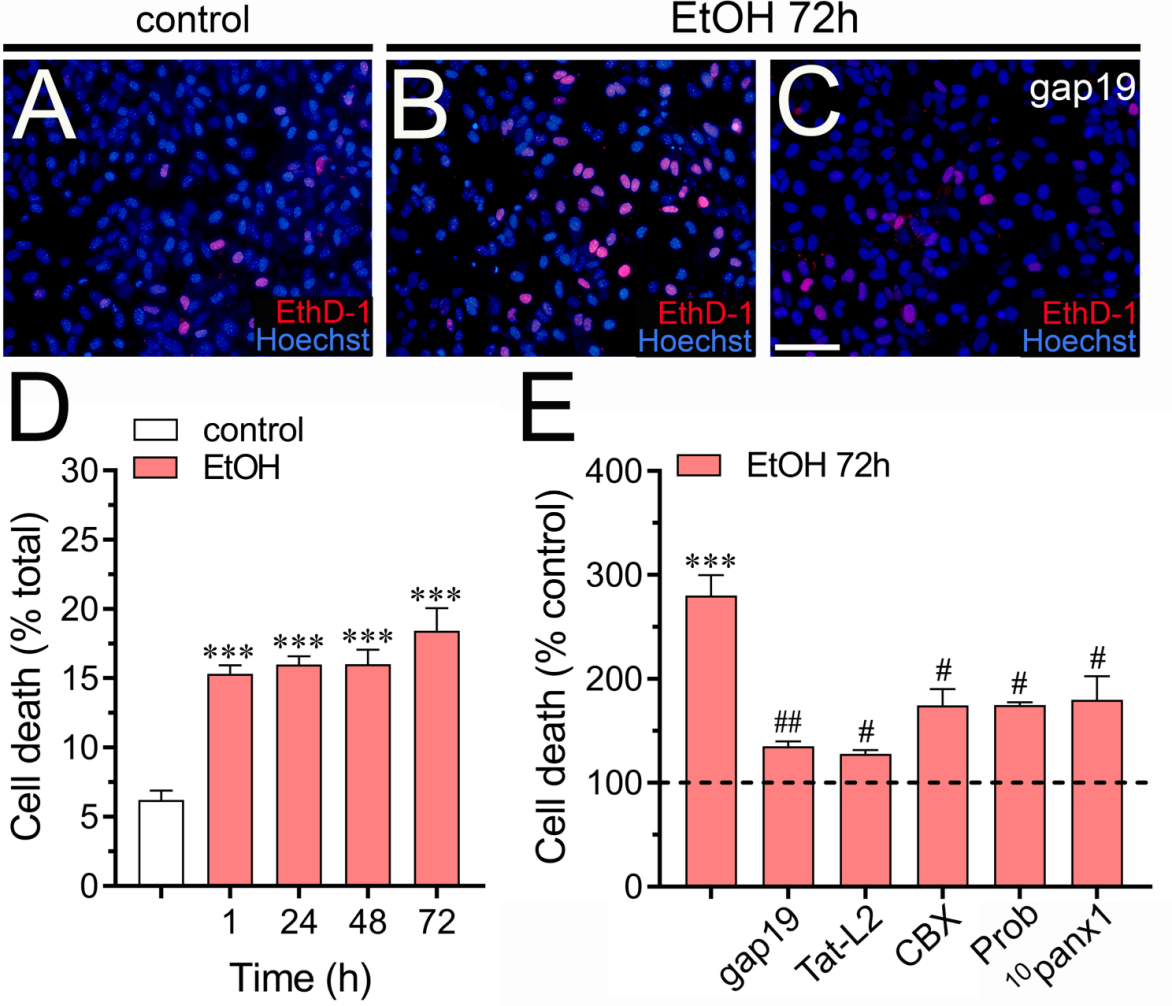
Hemichannels and pannexons contribute to ethanol-induced cell death in cultured astrocytes. (**A-C**) Representative fluorescence micrographs of Eth-D1 (red) uptake and Hoechst 33,342 nuclear staining (blue) by astrocytes under control conditions (**A**) or treated for 72 h with 100 mM ethanol alone (**B**) or in combination with 50 µM gap19 (**C**). (**D**) Quantitation of cell death (Eth-D1 staining) as a percentage of total cells (Hoechst 33,342) by astrocytes under control conditions (white bars) or treated for 1, 24, 48 or 72 h with 100 mM ethanol (red bars). **p < 0.001, ethanol treatment compared to control conditions (one-way ANOVA followed by Tukey’s post-hoc test). (**E**) Quantitation of cell death normalized to control conditions (dashed line) by astrocytes treated for 72 h with 100 mM ethanol alone (red bars) or in combination with the following pharmacological agents: 50 µM gap19, 50 µM Tat-L2, 5 µM CBX, 500 µM Prob or 50 µM ^10^panx1. ***p < 0.0005, ethanol compared to control; ^#^*p* < 0.05, ^##^*p* < 0.001; effect of pharmacological agents compared to ethanol treatment (one-way ANOVA followed by Tukey’s post-hoc test). Data were obtained from at least three independent experiments with three or more repeats each one. Calibration bar = 150 μm

To assess the potential impact of ethanol on astroglial survival, we examined the incorporation of EthD-1, a marker indicating loss of membrane integrity. Due to its large size, EthD-1 is only taken up by cells with disrupted membranes. Under control conditions, a few astrocytes show positive intracellular incorporation of EthD-1 (Fig. 7A-D). However, after 1 to 72 h of treatment with ethanol, approximately 15–18% of astrocytes took up EthD-1, indicating cell death (Fig. 7D). Similar findings were observed when Rhodamine B Dextran (10 kDa) was used, a dye that does not penetrate healthy cells (Supplementary Fig. 2A-C). Notably, the astrocyte death induced by 72 h of treatment with ethanol was significantly prevented by the Cx43 hemichannel blockers gap19 and Tat-L2 [93], while the Panx1 channel inhibitors ^10^panx1, CBX and probenecid provided slight protection (Fig. 7E). These results suggest that ethanol increases the inflammatory profile of astrocytes along with cell death by a mechanism that involves the activation of Cx43 hemichannels and Panx1 channels.

## Discussion

In this investigation, we documented the first evidence that ethanol triggers the activation of Cx43 hemichannels and Panx1 channels in cultured astrocytes. This escalated hemichannel/pannexon activity stems from a complex mechanism that involves the activation of TLR4, P2X7 receptors, IL-1β and TNF-α signaling, and the stimulation of p38 MAPK/iNOS-dependent pathways. The profound ethanol-triggered activation of hemichannels and pannexons led to significant elevations in NO synthesis, cytokine secretion, gliotransmitter discharge, and both heightened astrocyte reactivity and cellular damage.

By assessing Etd uptake, we demonstrated that ethanol increases the activity of Cx43 hemichannels and Panx1 channels in primary cortical astrocytes in a time- and concentration-dependent manner. A well-established mimetic peptide known for its ability to antagonize Cx43 hemichannel opening, namely gap19, significantly and markedly counteracted the ethanol-induced Etd uptake. Moreover, the inhibitory effects were similarly observed with the blockade of Panx1 channels using ^10^panx1 or probenecid, underscoring the substantial contributions of both Cx43 hemichannels and Panx1 channels to the ethanol-induced Etd uptake. These findings were validated through alternative pharmacological strategies (gap19, ^10^panx1, probenecid, La^3+^, CBX) and by utilizing specific siRNAs to interfere with the expression of both Cx43 and Panx1.

Few years ago, we reported that intermittent non-voluntary ethanol consumption boosts the activity of astrocytic Cx43 hemichannels and Panx1 channels in brain slices of adolescent rats [16]. However, the temporal range employed in that study to examine channel activity (1 to 9 weeks after ethanol exposure) did not allow us to determine whether ethanol directly affects astrocytes or if it acts indirectly through another mechanism that could progressively alter them (e.g., factors released by other cell types). The data obtained here supports the idea that ethanol directly affects astrocytes, exacerbating the activity of hemichannels and pannexons with significant negative consequences for astroglial function and survival. Significantly, the concentrations at which ethanol triggered these responses align with clinically relevant blood alcohol concentrations [BACs] linked to binge drinking (0.08% [∽ 17 mM]), severe alcohol intoxication (0.3% [∽ 65 mM]), coma (0.4% [∽ 87 mM]), and death (0.5% [∽ 109 mM]) [94]. It’s noteworthy that individuals with alcohol tolerance may exhibit higher blood alcohol concentrations (BACs) exceeding 0.55% and still manage to walk and perform tasks [95].

Importantly, ethanol-mediated activation of hemichannels and pannexons also occurred in HeLa cells expressing exogenous Cx43^EGFP^ or Panx1^EGFP^, but not in parental HeLa cells. This suggests that the dye uptake induced by ethanol relies entirely on the specific activation of these channels when they are transfected into cells lacking endogenous expression of Cx43 or Panx1. Consistent responses between astrocytes and heterologous expression systems have been described before for other pathological stimuli that augment hemichannel and pannexon activity, including hypoxia-reoxygenation, metabolic inhibition and FGF-1 [96–100].

How does ethanol trigger the activity of Cx43 hemichannels in astrocytes? Seminal studies from Guerri’s Laboratory revealed that ethanol induces prolonged activation of astrocytes through TLR4/IL-1RI signaling, leading to the release of IL-1β/TNF-α and the activation of p38 MAPK and iNOS [20, 21]. Both IL-1β and TNF-α, along with their associated downstream signaling pathways, including p38 MAPK and iNOS, have been implicated in the opening of Cx43 hemichannels in astrocytes subjected to various pro-inflammatory conditions [35, 81, 92]. Coherent with these findings, we demonstrated that ethanol enhances astroglial production of IL-1β and TNF-α, and blocking TLR4 or IL-1β/TNF-α signaling significantly attenuated the ethanol-induced Etd uptake. Of note, ^10^panx1 but not gap19 elicited an additive reduction in the ethanol-induced Etd uptake and glutamate release when astrocytes were co-treated with the TLR4 inhibitor TAK-242. These results suggest that ethanol-induced opening of Cx43 hemichannels and subsequent glutamate release involve TLR4 receptors and likely their downstream signaling.

IL-1β and TNF-α boost the activity of astrocytic Cx43 hemichannels by activating a p38 MAPK-mediated pathway and inducing NO-dependent S-nitrosylation of Cx43 [54, 81]. We observed that ethanol elevates the production of NO by astrocytes. Interestingly, inhibiting both p38 MAPK and iNOS significantly decreased the ethanol-induced Etd uptake, implying that both pathways are downstream of TLR4-mediated activation of Cx43 hemichannels in ethanol-treated astrocytes. Moreover, ethanol increased NO production via TLR4, and this response was predominantly sustained by the downstream activation of Cx43 hemichannels but not Panx1 channels. These results harmonize with previous studies documenting that NO production may be linked to the persistent opening of Cx43 hemichannels in astrocytes [34, 92].

Astrocytes exposed to ethanol release ATP and glutamate [101, 102], which, at high concentrations, can cause excitotoxicity [103–105]. In this investigation, we found that inhibiting Panx1 channels, but not Cx43 hemichannels, completely suppressed the ethanol-induced release of ATP. Interestingly, the opposite pattern was observed for glutamate release, implicating Cx43 hemichannels but Panx1 channels. This suggests that while ethanol induces the opening of both channels, it occurs through different activation pathways, resulting in distinct gliotransmitter releases. As discussed earlier, TLR4 and IL-1β/TNF-α/p38 MAPK/iNOS-dependent signaling appear to be involved in Cx43 hemichannels activation and subsequent glutamate release.

Our data indicate that the ethanol-induced opening of Panx1 channels occurs via P2X7 receptors, leading to the specific release of ATP but not glutamate. In fact, ethanol-induced Etd uptake was partially decreased by blocking P2X7 receptors, and ^10^panx1 did not cause any additional reduction to this inhibitory effect. Previous evidences indicates that stimulation of P2X7 receptors not only opens a non-selective cation channel but also induces the passage of large positive molecules, including Etd and YO-PRO1 [106]. The exact mechanisms governing this phenomenon are not fully understood, but evidence suggests two possible pathways: a direct entry through the P2X7 pore itself, and an indirect pathway triggered by P2X7 receptor activation involving additional proteins [107]. One of the pathways associated with the P2X7 receptor-activated pore involves the opening of Panx1 channels [73, 108, 109]. Here, we observed that blocking Panx1 hemichannels or P2X7 receptors completely inhibits the ethanol-induced release of ATP and Etd uptake. Thus, it is highly probable that the initial activation of Panx1 hemichannels is induced by P2X7 receptors through protein–protein interactions, likely involving the proline-rich region situated at the carboxyl-terminal domain of the P2X7 receptor [109]. This is in agreement with the fact that ethanol stimulates P2X7 receptors [110, 111]. Furthermore, long-lasting production of IL-1β induced by ethanol was also strongly reduced upon blockade of Panx1 channels or P2X7 receptors. The latter is coherent with other reports showing that Panx1 hemichannels and P2X7 receptors participate in the maturation of IL-1β in the context of the inflammasome activation in astrocytes [90, 112].

The observation that ethanol induces a distinct release of ATP or glutamate depending on the large-pore channel-forming protein (Cx43 or Panx1) raises questions. One possibility is that, despite both channels being permeable to Etd, they differ in their roles in releasing ATP and glutamate in our system. This may seem paradoxical, but recent studies indicate that hemichannels and pannexons are not freely permeable non-selective pores; instead, they selectively allow certain molecules in an isoform-specific manner [113–115]. Thus, the uptake of fluorescent dyes may not accurately reflect permeability to ions or small biologically relevant molecules, a characteristic that might even change under pro-inflammatory conditions [69]. Similar differential patterns of gliotransmitter release for Cx43 hemichannels and Panx1 channels have been documented before in astrocytes subjected to α-synuclein or prenatal LPS treatment [35, 92].

Mounting evidence has demonstrated that ethanol activates the NF-κB pathway in astrocytes, inducing astroglial reactivity and substantial changes in their inflammatory profile [17, 20, 116]. In this study, we observed a significant increase in both total GFAP and nuclear levels of NF-κB p65 in astrocytes treated with ethanol. Similar findings have been reported in vitro and ex vivo experimental models of ethanol exposure [15, 16, 116, 117]. Indeed, neonatal ethanol exposure leads to an increase in GFAP expression, which is rather confined to specific areas, suggesting that this is caused by increased neuronal damage or the release of pro-inflammatory cytokines by microglia [117]. NF-κB activation could potentially be responsible for the increased expression of GFAP in our system. Previous evidence suggests that NF-κB might enhance GFAP transcription by binding to its promoter [118]. Importantly, we also found that ethanol-induced increase in total GFAP and nuclear levels of NF-κB p65 were accompanied by a persistent decrease in astrocytic survival. Notably, we found that ethanol-induced alterations in GFAP and NF-κB p65 levels, as well as astroglial survival, were robustly prevented by blocking Cx43 hemichannels, while inhibition of Panx1 channels provided slight protection. These findings align with previous research demonstrating that inhibition of hemichannels could mitigate astroglial reactivity and death triggered by various pro-inflammatory conditions [35, 92, 119].

The mechanisms underlying the large-pore channel-dependent alterations in ethanol-treated astrocytes could be manifold. The observed necrosis in ethanol-induced astrocytes might result from cell swelling and plasma membrane breakdown caused by the uncontrolled entry of Na^+^ and Cl^−^ through hemichannels and pannexons [120, 121]. Additionally, since these channels directly or indirectly contribute to the increase in [Ca^2+^]_i_, their persistent activation could lead to Ca^2+^ overload, resulting in the perturbation of astrocytes; namely, changes in the production of gliotransmitters, cytokines and NO, as well as increased reactivity (GFAP and NF-κB overexpression).

## Conclusion

In conclusion, our study reveals a new mechanism by which ethanol impairs astrocyte function, involving the sequential stimulation of inflammatory pathways that further increase the opening of Cx43 hemichannels and Panx1 channels. We propose that exacerbated hemichannel and pannexon activity in astrocytes may trigger the release of high amounts of potentially toxic molecules for neurons, such as ATP and glutamate. Understanding this mechanism offers a promising avenue for developing novel pharmacological interventions designed to safeguard astrocyte integrity and promote neuronal resilience against the complexities of various alcohol use disorders.

## Materials and methods

### Reagents and antibodies

HEPES, water (W3500), DNAse I, poly-L-lysine, L-N6, SB203580, anti-Cx43 polyclonal antibody, anti-GFAP monoclonal antibody, carbenoxolone (CBX), ATP, TAK-242, La^3+^, A74003, oxidized ATP (oATP), glutamate determination kit probenecid (Prob) were purchased from Sigma-Aldrich (St. Louis, MO, USA). Fetal bovine serum (FBS) was obtained from Hyclone (Logan, UT, USA). Penicillin, streptomycin, Trypsin 10X, Hank’s solution, ATP determination kit, Dulbecco’s Modified Eagle Medium (DMEM), Phosphate-Buffered Saline (PBS), Etd bromide (10 mg/mL), DAF-FM, Hoechst 33,342, diamidino-2-phenylindole (DAPI), ethidium homodimer-1 (EthD-1), wheat germ agglutinin (WGA) Alexa Fluor™ 555 conjugate, goat anti-mouse Alexa Fluor 488/555 and goat anti-rabbit Alexa Fluor 488/555 were purchased from Thermo Fisher Scientific (Waltham, MA, USA). Anti-Panx1 monoclonal antibody ab124131 was purchased from Abcam (Cambridge, UK), whereas anti-Panx1 monoclonal antibody (D9M1C) and anti-NF-κB p65 antibody (D14E12) was obtained from Cell Signaling (Danvers, MA, USA), respectively. Normal goat serum (NGS) was purchased from Zymed (San Francisco, CA, USA). Anti-Cx43 monoclonal antibody (610,061) was obtained from BD Biosciences (Franklin Lakes, NJ, USA). IL-1β and TNF-α were obtained from Roche Diagnostics (Indianapolis, MI, USA). A soluble form of the TNF-α receptor (sTNF-αR1) and a recombinant receptor antagonist for IL-1β (IL-1ra) were from R&D Systems (Minneapolis, MN). Horseradish peroxidase (HRP)-conjugated anti-rabbit IgG was purchased from Pierce (Rockford, IL, USA). Gap19 (KQIEIKKFK, intracellular loop domain of Cx43), gap19^I130A^ (KQAEIKKFK, negative control), Tat-L2 (YGRKKRRQRRRDGANVDMHLKQIEIKKFKYGIEEHGK, second intracellular loop domain of Cx43) and ^10^panx1 (WRQAAFVDSY, first extracellular loop domain of Panx1) peptides were obtained from Genscript (New Jersey, USA).

### Animals

Animal experimentation was conducted in accordance with the guidelines for the care and use of experimental animals of the US National Institutes of Health (NIH), the ad hoc committee of the Chilean government (ANID), the Bioethics Committee of the Pontificia Universidad Católica de Chile (PUC) (n°: 200,605,010) and the European Community Council Directives of November 24th, 1986. C57BL/6 (PUC) mice of 8–9 weeks of age were housed in cages in a temperature-controlled (24 °C) and humidity-controlled vivarium under a 12 h light/dark cycle (lights on 8:00 AM), with *ad libitum* access to food and water.

### Cell cultures

Astrocytes: astroglial cell primary cultures were prepared from the cortex of postnatal day 2 (P2) mice as previously described [79]. Briefly, brains were removed, and cortices were dissected. Meninges were carefully peeled off and tissue was mechanically dissociated in Ca^2+^ and Mg^2+^ free Hank’s balanced salt solution (CM-HBSS) with 0.25% trypsin and 1% DNase. Cells were seeded onto 35-mm plastic dishes (Corning, NY, USA) or onto glass coverslips (Fisher Scientific, Waltham, MA, USA) placed inside 24-well plastic plates (Corning, NY, USA) at the density of 5 × 10^5^ cells/dish or 1 × 10^5^ cells/well, respectively, in DMEM, supplemented with penicillin (5 U/ml), streptomycin (5 µg/ml), and 10% FBS. Cells were grown at 37 °C in a 5% CO2/95% air atmosphere at nearly 100% relative humidity. Following 8–10 days in vitro (DIV), 1 µM AraC was added for 3 days to suppress the proliferation of microglia. Medium was changed twice a week and cultures were used after 3 weeks. At that stage, these cultures contained > 97% GFAP + cells. No neurons were detected as judged by MAP2 and NeuN staining.

HeLa cells: Parental HeLa cells knock-out for Cx45 (HeLa-KO45) were used, to ensure no endogenous expression of this connexin. HeLa cells were stably transfected with mouse Cx43^EGFP^ or Panx1^EGFP^, with EGFP fused to the C-terminus of these proteins, as previously described [75, 76]. Cells were cultured in low glucose DMEM media supplemented with 10% FBS as well as 50 U/ml penicillin and streptomycin at 37◦C in a 5% CO_2_/95% O_2_ atmosphere. Cells were selected by their resistance to the antibiotic geneticin (G418, maintained with 1 mg/ml in the medium). The culture medium was replaced every other day.

### Treatments

Astrocytes or HeLa-cells were treated for 0, 1, 24, or 48 h with 0, 1, 10, 25, 50, or 100 mM of EtOH. The following pharmacological agents were pre-incubated 1 h prior and co-incubated with 100 mM ethanol before experiments: mimetic peptides against Cx43 hemichannels (gap19, 50 µM) and pannexin1 (Panx1) channels (^10^panx1, 50 µM), Probenecid (Prob, pannexin channel blocker, 200 µM), TAK-242 (TLR4 inhibitor, 0.5 µM), sTNF-αR1 (soluble form of the receptor that binds TNF-α), IL-1ra (IL-1β receptor endogenous blocker), SB203580 (p38 MAP kinase inhibitor, 1 µM), L-N6 (iNOS inhibitor, 1 µM), oATP (general P2X receptor blocker, 200 µM), A740003 (P2X7 receptor blocker, 200 nM). To obtain conditioned media (CM) from astrocytes, supernatants were collected, filtered (0.22 μm), and stored at -20 °C.

### siRNA transfection

siRNA duplexes against mouse Cx43 or Panx1 were predesigned and obtained from Origene (Rockville, MD). siRNA (10 nM) was transfected with Oligofectamine (Invitrogen) according to the Origene application guide for Trilencer-27 siRNA. Negligible cell death was detected after transfection (data not shown). Sequences for siRNAs against mouse Cx43 and Panx1 were: siRNA-Cx43: rGrCrArGrUrGrCrArCrArUrGrUrArArCrUrArArUrUrUrATT and siRNA-Panx1: rArGrArArCrArUrArArGrUrGrArGrCrUrCrArArArUrCrGTA, respectively. Transfection experiments were performed 48 h before the treatment with 100 mM EtOH for 24 h.

### Dye uptake and time-lapse fluorescence imaging

For dye uptake experiments in astrocytes, they were plated on 12 mm glass coverslips and, after two weeks of culture, were washed twice in Hank´s balanced salt solution. Then, astrocytes were incubated at room temperature with recording solution (in mM): 148 NaCl, 5 KCl, 1.8 CaCl_2_, 1 MgCl_2_, 5 glucose, and 5 HEPES, pH 7.4, containing 5 µM Etd and mounted on the stage of an Olympus BX 51W1I upright microscope with a 40x water immersion objective for time-lapse imaging. Images were captured by a Retiga 1300I fast-cooled monochromatic digital camera (12-bit) (Qimaging, Burnaby, BC, Canada) controlled by imaging software Metafluor software (Universal Imaging, Downingtown, PA) every 30 s (exposure time = 0.5 s; excitation and emission wavelengths were 528 nm 598 nm, respectively). For dye uptake experiments in HeLa cells, they were first seeded on 25 mm glass coverslips and used when they reached 70–80% confluency. Then, they were bathed with recording Krebs solution (in mM): 118 NaCl, 4.7 KCl, 3 CaCl_2_, 1.2 MgCl_2_, 10 glucose, 20 HEPES, 9.9 Tris; pH 7.4; containing 5 µM DAPI. Fluorescence intensity images were recorded in cells that were selected for having a fluorescent label, indicating that they express Cx43^EGFP^ or Panx1^EGFP^. The images were taken with a NIKON Eclipse Ti inverted microscope (Japan) every 15 s for 5 min per condition. Nikon software (NIS Elements Advanced Research) was used for off-line image analysis. For astrocytes and HeLa cells, the fluorescence intensity recorded from at least 20 regions of interest (representing 20 cells per cultured coverslip) was calculated with the following formula: Corrected total cell fluorescence = Integrated Density – ([Area of selected cell] x [Mean fluorescence of background readings]). The mean slope of the relationship over a given time interval (ΔF/ΔT) represents the dye uptake rate and was calculated with regression lines that were fitted to points before and after the various experimental conditions using Microsoft Excel (Seattle, WA, USA). The mean values of slopes were plotted using GraphPad Prism 7 software (La Jolla, California, USA) and expressed as AU/min. At least three replicates (four sister cultured coverslips) were measured in each independent experiment. In some experiments, cultured astrocytes were pre-incubated with Cx43 and/or Panx1 channel blockers for 15 min before and during the time-lapse experiments of Etd uptake: gap19 (50 µM), La^3+^ (200 µM), CBX (5 µM), ^10^panx1 (50 µM), gap19^I130A^ (50 µM) or Prob (200 µM).

### Western blot analysis

Astrocytes were rinsed twice with PBS (pH 7.4) and harvested by scraping with a rubber policeman in ice-cold PBS containing 5 mM EDTA, Halt (78,440) and M-PER protein extraction cocktail (78,501) according to the manufacturer instructions (Pierce, Rockford, IL, USA). The cell suspension was sonicated on ice. Proteins were measured using the Bio-Rad protein assay. Aliquots of cell lysates (100 µg of protein) were resuspended in Laemli’s sample buffer, separated in an 8% sodium dodecyl sulfate polyacrylamide gel electrophoresis (SDS-PAGE) and electro-transferred to nitrocellulose sheets. Nonspecific protein binding was blocked by incubation of nitrocellulose sheets in PBS-BLOTTO (5% nonfat milk in PBS) for 30 min. Blots were then incubated with primary antibody at 4 °C overnight, followed by four 15 min washes with PBS. Then, blots were incubated with HRP-conjugated goat anti-rabbit antibody at room temperature for 1 h and then rinsed four times with PBS for 15 min. Immunoreactivity was detected by enhanced chemiluminescence (ECL) detection using the SuperSignal kit (Pierce, Rockford, IL) according to the manufacturer´s instructions.

### Immunofluorescence and confocal microscopy

Astrocytes grown on coverslips were fixed at room temperature with 2% paraformaldehyde (PFA) for 30 min and then washed three times with PBS. They were incubated three times for 5 min in 0.1 M PBS-glycine, and then in 0.1% PBS-Triton X-100 containing 10% NGS for 30 min. Cells were incubated with anti-GFAP monoclonal antibody (Sigma, 1:400), anti-NF-κB p65 monoclonal antibody (Cell signaling, 1:600), anti-Cx43 polyclonal antibody (SIGMA, 1:400) or anti-Panx1 monoclonal antibody (ABCAM 1:400) diluted in 0.1% PBS-Triton X-100 with 2% NGS at 4 °C overnight. After five rinses in 0.1% PBS-Triton X-100, cells were incubated with goat anti-mouse IgG Alexa Fluor 355 (1:1000) or goat anti-rabbit IgG Alexa Fluor 488 (1:1000) at room temperature for 50 min. After several rinses, coverslips were mounted in DAKO fluorescent mounting medium and examined with a Zeiss Axio Observer D.1 Inverted Microscope with a Solid-State Colibri 7 LED illuminator and 60x oil immersion objective or with a confocal system Nikon Ti2-E inverted microscope and a 60 × (1.4 NA) Plan Apo oil immersion objective. In a set of experiments, the plasma membrane of astrocytes was stained with Wheat-germ agglutinin (WGA) labeled with Alexa Fluor 555 (5 µg/mL) for 15 min at 37ºC before fixation with PFA. Nuclei were stained with DAPI or Hoechst 33,342. To assess the fluorescent intensity of Cx43 or Panx1 in the plasma membrane area labeled with WGA, stacks of consecutive confocal images were taken with a confocal system Nikon Ti2-E inverted microscope and a 60 × (1.4 NA) Plan Apo oil immersion microscope objective at 200 nm intervals. Images were acquired sequentially with three lasers (in nm: 408, 488 and 543). Image analysis and Manders’ coefficients were performed with ImageJ software. Cx43 or Panx1 signal intensity in both plasma membrane and cytoplasm was calculated with the following formula: Corrected cell stain fluorescence = Integrated Density – ([Area of selected cell] x [Mean fluorescence of background readings]). At least 50 cells for each treatment randomly taken from three independent experiments. Images shown in each figure are all central and cross the nucleus visualized by DAPI staining.

### NO production

Astrocytes plated on glass coverslips were loaded with 5 µM DAF-FM in DMEM without serum at 37 °C for 45 min and then washed three times in Locke’s solution in mM: 154 NaCl, 5.4 KCl, 2.3 CaCl_2_, 5 HEPES, pH 7.4; followed by de-esterification at 37 °C for 15 min. Coverslips were then mounted in an Olympus BX 51W1I upright microscope with a 40x water immersion objective. Pictures were captured at 495 excitation wavelength and the emission was filtered at 505–550 nm using a Retiga 1300I fast-cooling monochrome digital monochrome camera (12-bit; Q Imaging, Burnaby, BC, Canada) controlled by Metafluor imaging software (Universal Imaging, Downingtown, PA, USA). Fluorescence intensity captured from at least 25 regions of interest (representing 25 cells per cultured coverslip) was calculated with the following formula: Corrected total cell fluorescence = Integrated Density – ([Area of selected cell] x [Mean fluorescence of background readings]).

### IL-1β and TNF-α determination assay

IL-1β and TNF-α were determined in the astrocyte CM. Samples were centrifuged at 14.000 g for 40 min. Supernatants were collected and protein content was assayed by the BCA method. IL-1β and TNF-α levels were determined by sandwich ELISA, according to the manufacturer’s protocol (eBioscience, San Diego, CA, USA). For the assay, 100 µl of samples were added *per* ELISA plate well and incubated at 4 °C overnight. A calibration curve with recombinant cytokine was included. Detection antibody was incubated at room temperature for 1 h and the reaction developed with avidin–HRP and substrate solution. Absorbance was measured at 450 nm with reference to 570 nm with the microplate reader Synergy HT (Biotek Instruments).

### Measurement of extracellular ATP and glutamate concentration

Extracellular ATP in CM of astrocytes was measured using a luciferin/luciferase bioluminescence assay kit (Sigma-Aldrich), while extracellular levels of glutamate were determined using an enzyme-linked fluorimetric assay (Sigma-Aldrich). Cells were lysed with a Tris-buffered solution containing 1% TritonX-100 and supernatants of whole-cell lysates were used for measurements of intracellular ATP and glutamate levels. The amounts of ATP and glutamate in the samples were calculated from standard curves and normalized for the protein concentration using the Bio-Rad protein assay.

### Astrocyte death quantification

Astrocyte membrane breakdown was evaluated using the cell-impermeant viability indicator Ethidium homodimer-1 (EthD-1, 856.75 Da) from ThermoFisher (#E1169). EthD-1 does not enter healthy cells upon stimulation of Cx43 or Panx1 hemichannels with divalent cation-free solution or BzATP, respectively (data not shown). Briefly, cells were incubated with Hank´s balanced salt solution with EthD-1 (5 µM) and Hoechst 33,342 (1 µM) at 37ºC for 15 min and then washed three times in PBS. Hoechst 33,342 and EthD-1 imaging involved data acquisition (emission at 350 and 528 nm, respectively) at 461 and 628 nm excitation wavelengths, respectively, using a Zeiss Axio Observer D.1 Inverted Microscope with a Solid-State Colibri 7 LED illuminator and 20x objective. In some experiments, the same experimental approach was performed using Rhodamine B Dextrane (Rdex, 10 kDa) instead EthD-1. The quantification of cell death was expressed as the percentage of cells that incorporated EthD-1 (red cells) in relation to the total cell population (blue cells) in each image captured. A total of 15 images (20–30 cells per image) for each experiment were analyzed using the ImageJ software.

### Data analysis and statistics

Detailed statistical results were included in the figure legends. Statistical analyses were performed using GraphPad Prism (version 7, GraphPad Software, La Jolla, CA). Normality and equal variances were assessed by the Shapiro-Wilk normality test and Brown-Forsythe test, respectively. Unless otherwise stated, data that passed these tests were analyzed by unpaired t-test in case of comparing two groups, whereas in case of multiple comparisons, data were analyzed by one or two-way analysis of variance (ANOVA) followed, in case of significance, by a Tukey’s post-hoc test. A probability of *P* < 0.05 was considered statistically significant.

### Electronic supplementary material

Below is the link to the electronic supplementary material.

Supplementary Material 1

Supplementary Material 2

Supplementary Material 3

## Data Availability

The datasets used and/or analyzed during the current study are available from the corresponding author upon reasonable request.

## Abbreviations

Cx43: Connexin 43
CBX: carbenoxolone
DAPI: diamidino-2-phenylindole
DMEM: Dulbecco’s Modified Eagle Medium
EGFP: Enhanced green fluorescent protein
Etd: Ethidium
EthD-1: Ethidium homodimer-1
FBS: Fetal bovine serum
GFAP: glial fibrillary acidic protein
iNOS: inducible nitric oxide synthase
NO: nitric oxide
oATP: oxidized ATP
p38 MAPK: p38 mitogen-activated protein kinase
Panx1: Pannexin-1
PBS: Phosphate-Buffered Saline
TLR4: Toll-like receptor 4
WGA: wheat germ agglutinin
