# Supplementary figures and images for: Cx43 hemichannels and panx1 channels contribute to ethanol-induced astrocyte dysfunction and damage
Source: Biol Res. 2024 Apr 4;57:15. doi: 10.1186/s40659-024-00493-2 (PMC10996276; doi:10.1186/s40659-024-00493-2)

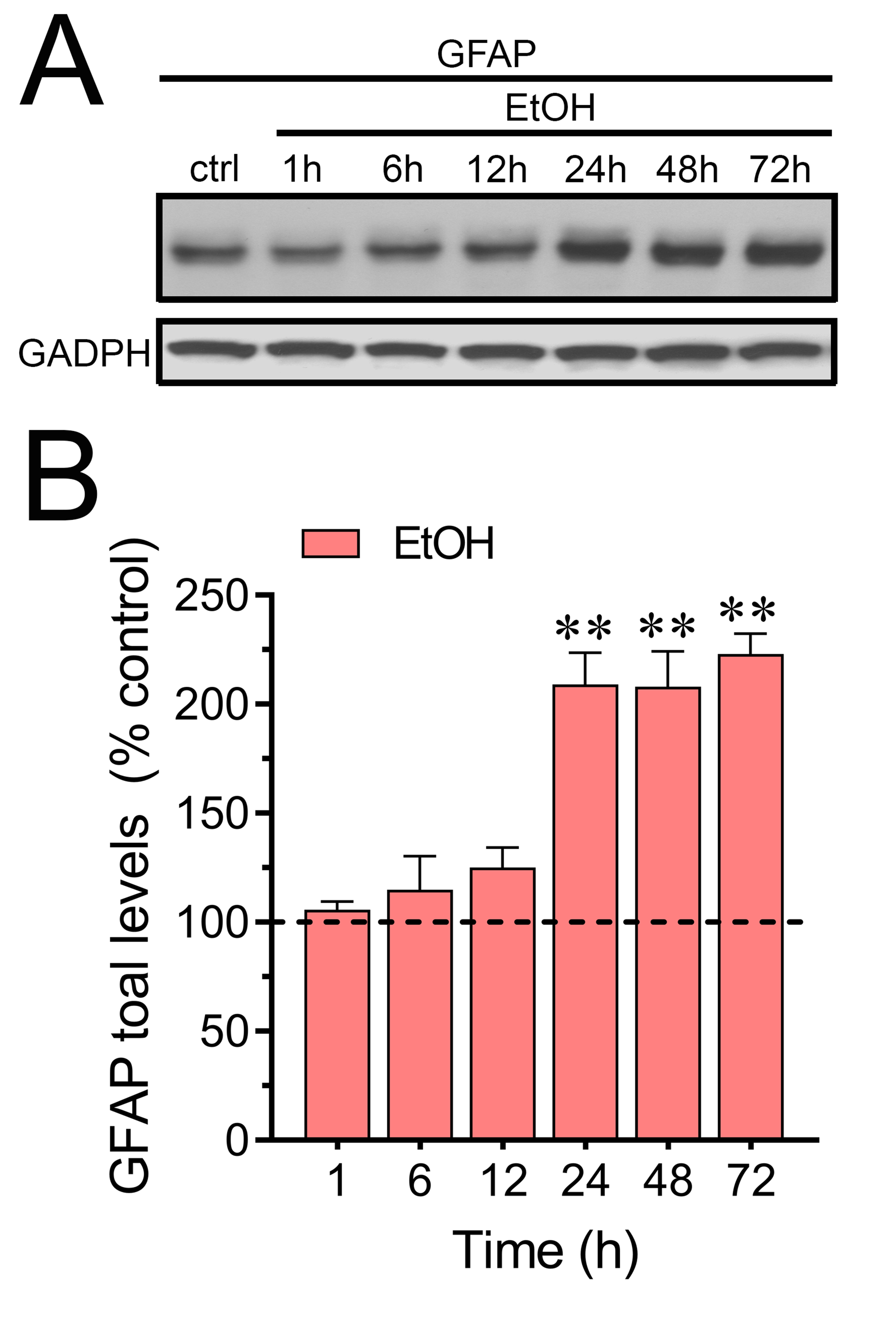

Supplement: Supplementary file 1 — Supplementary Material 1 [file 40659_2024_493_MOESM1_ESM.tif]

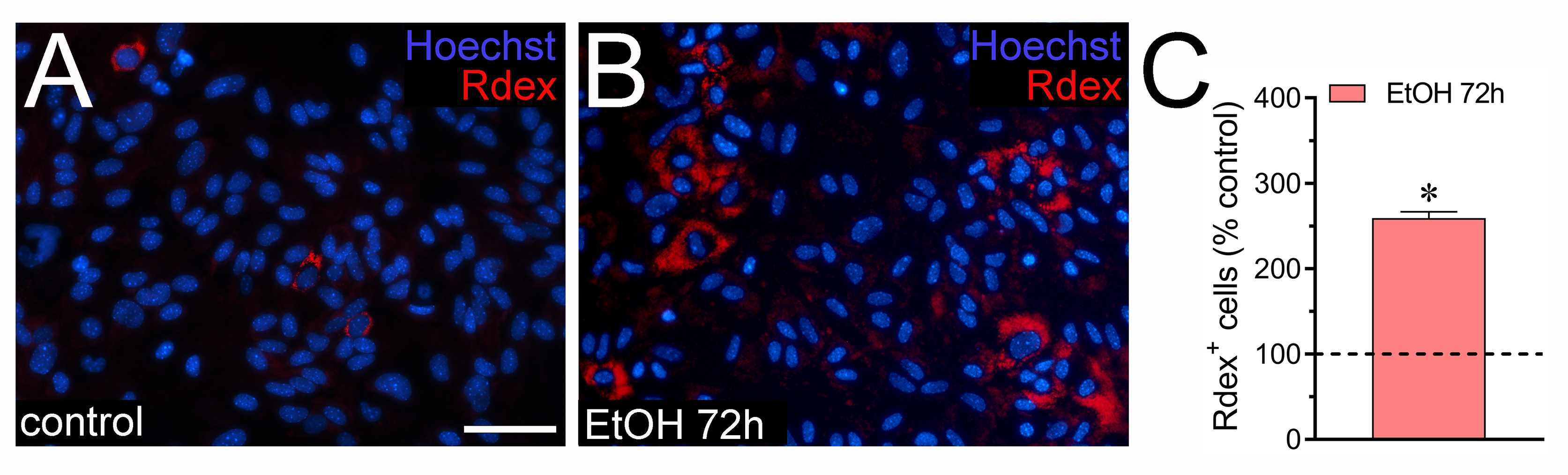

Supplement: Supplementary file 2 — Supplementary Material 2 [file 40659_2024_493_MOESM2_ESM.tif]

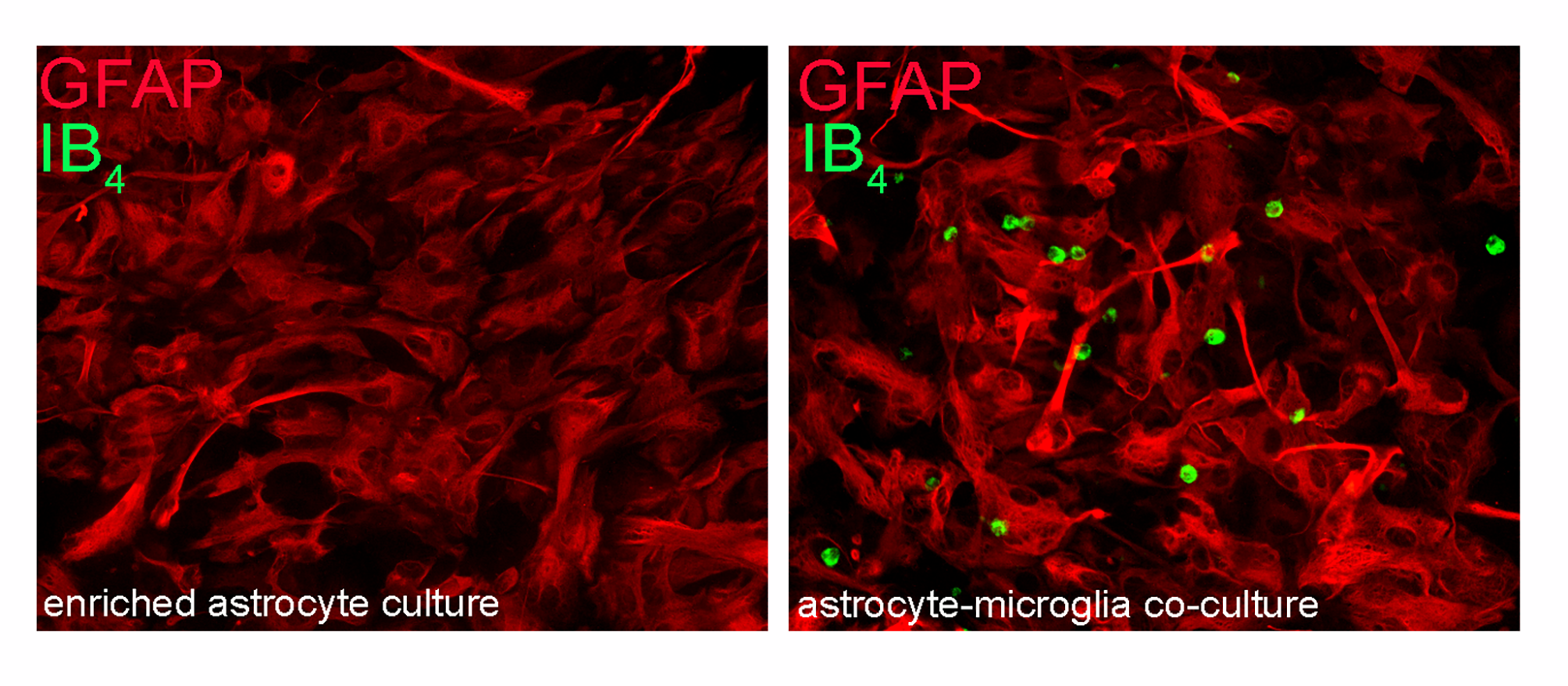

Supplement: Supplementary file 3 — Supplementary Material 3 [file 40659_2024_493_MOESM3_ESM.tif]
